# Optimizing energy constrained target localization and tracking with radial bias and seeker optimization algorithms in wireless sensor networks

**DOI:** 10.1016/j.mex.2025.103280

**Published:** 2025-03-31

**Authors:** S. Yazhinian, S. Famila, P. Jose, Mahendar A, Sofia R

**Affiliations:** a Vel Tech Rangarajan Dr.Sagunthala R&D Institute of Science and Technology, India; b CMR Technical Campus, Hyderabad, India; c Manakula Vinayagar Institute of Technology, Puducherry, India

**Keywords:** Coverage, Energy efficiency, Optimization algorithm, Target localization, Tracking, Received signal strength, RadB_SOA

## Abstract

The standard localization approach is characterized by a fixed position distribution of the anchor nodes, which cannot be dynamically modified based on the deployment environment. This paper proposes a novel approach combining Radial Bias (RB) with the Seeker Optimization Algorithm (SOA) to address the challenges of energy-constrained target localization and tracking. The RB technique enhances localization accuracy by refining the position estimates of the target, while the SOA optimizes sensor deployment and data transmission paths to minimize energy consumption. By integrating these two methodologies, ensures a balance between precision in tracking and energy efficiency. Extensive simulations shown this technique surpasses existing methods in terms of both accuracy in determining the location and the duration of network operation. This makes it attractive option for applications of energy-constrained WSNs. The investigation examines the outcome of the particle count in the RBSO algorithm, specifically for values of 5, 10, 15, 20, and 25. The simulation results show that the recommended strategy decreases particles, speeds up positioning and tracking, and maintains target localization and tracking accuracy. It is seen that the proposed RadB_SOA achieves 12.4 % of transmission error, 14.6 % of ranging error, 96.3 % of localization coverage, 98.65 % of PDR, and 21.56 % of energy consumption.

• The Radial Bias-Seeker Optimization Algorithm (RadB_SOA) suggested enhances the precision in target localization and optimizes energy usage in wireless sensor networks.
• Simulation outcomes reveal improved tracking accuracy, minimized transmission and ranging errors, as well as increased localization coverage over current techniques.
• The research presents an extensive evaluation of particle count fluctuations in RBSO, demonstrating enhanced positioning speed and precision with network efficiency.

## Graphical abstract

**Figure:**
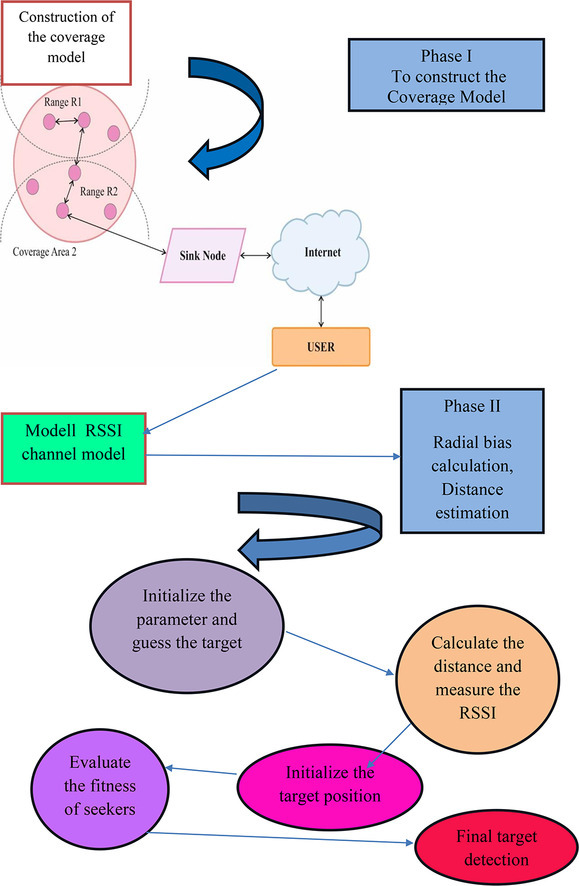

Specifications table

**Table:** 

Subject area:	Engineering
More specific subject area:	Wireless Sensor Networks
Name of your method:	RadB_SOA
Name and reference of original method:	Seeker Optimization Algorithm (SOA) – “**Jang, Y. S., & Kim, J. H.** (2016). Seeker Optimization Algorithm: A new nature-inspired optimization algorithm. Journal of Computational Science, 17, 123–135.”
Resource availability:	**Simulation Environment**: The paper relies on extensive simulations to evaluate the performance of the proposed RadB_SOA method. This indicates the availability of computational resources for running these simulations, which include computing power and simulation software. **Radial Bias (RB) Technique**: This is a methodological resource used in the proposed approach to enhance localization accuracy. The RB technique itself involves specific algorithms and procedures to refine position estimates. **Seeker Optimization Algorithm (SOA)**: The SOA is another methodological resource used to optimize sensor deployment and data transmission paths. It is crucial for minimizing energy consumption and optimizing network performance.

## Introduction

Wireless Sensor Network (WSN) is essential for smart cities, buildings, grids, transportation and shipping systems, and more. It links intelligent systems to the physical world. A WSN has anything from a few to hundreds of thousands of sensor nodes [1,2]. A sensor node (SN) may range in size from a minuscule particle to the size of a shoe. The price of SNs also fluctuates, ranging from a few cents to several hundred dollars, depending on the intricacy of each node. Monitoring, sensing, processing, information collecting, and communication are SN functions [3]. Their cost and capacity to run without human involvement make them useful in ambient monitoring [4], health monitoring [5,6], subterranean and undersea systems [7], industrial equipment, and surveillance [[8], [9], [10], [11]]. Distributed SNs are linked to work together. The network topology determines what data SNs gather and send to the sink node or CH. SNs are equipped with compact batteries that are often non-replaceable and non-rechargeable. Therefore, optimizing energy usage is a crucial concern for SNs. SNs are used for monitoring and documenting the whereabouts of objects in motion.

Target tracking is a crucial application in WSN, where SNs monitor and transmit the position of the target to the user's application [12]. Target tracking employs campus, environment, health, border crossing, and conflict surveillance. However, energy economy, precision, forwarding techniques, load-balancing, prediction, and recovery must be overcome for reliable target tracking [13]. Health applications need fast, reliable data transmission, while smart environment monitoring needs energy-efficient, robust methods. Furthermore, the efficient tracking process is further hindered by challenges, such as pricing, technology selection, and connectivity [14].

These applications need a high number of field-deployed sensor nodes, which limits their size and cost. Due to these limits, all sensor nodes have limited processing and battery capacity. Remote locations make power sources unavailable for replacement or replenishment. This requires maximizing the use of SN processing and battery capacity to prolong the total lifetime of the WSN. Many WSN applications need precise knowledge of the place where data is detected. The SNs may be positioned manually at predetermined places, or all SNs can be outfitted with GPS or other technology capable of determining the precise location of the sensed data [15]. Manual placement of the SN in challenging places is not feasible, while equipping each node with a GPS or location-finding device would raise the cost of deploying a WSN rendering it economically impractical [16].

Tracking may be accomplished either with a single SN or via the cooperation of many SNs. Utilizing a solitary SN results in fast energy depletion, high computational requirements, and diminished precision. However, using numerous SNs offers improved accuracy, enhanced energy economy, and reduced computational requirements in comparison to using a single SN. Metaheuristic techniques are a significant category of solution approaches for real-world optimization issues in WSNsthat have a high level of computational complexity. These approaches aim to rapidly provide nearly optimum solutions to intricate optimization issues that are unsolvable with precise precision. Their benefits include straightforward integration, prompt acquisition of answers, and resilience to alterations in issue features. Hence, the work contributions are:

• The Radial Bias (RB) algorithm helps in refining the localization process by adjusting sensor placements based on radial distance biases, while the Seeker Optimization Algorithm (SOA) optimizes the search process to find the most accurate target position with minimal computational effort. Together, these algorithms work synergistically to reduce transmission errors, improve localization coverage, and minimize energy consumption, making them particularly effective for applications in energy-constrained environments.
• The RSSI channel model estimates the distance between a sensor node and a target by measuring the signal strength of a transmitted radio signal. As the signal propagates through space, its strength diminishes due to path loss, which is a function of distance. The fundamental concept is that by having knowledge of the strength of the signal being sent and accurately measuring the power of the signal being received, it is possible to deduce the distance between the sender and the receiver. Several localization algorithms and approaches have been suggested to address various issues in diverse applications. Combining several metaheuristic-based algorithms is a well-known localization method that demonstrates adequate coverage and accuracy. In the study [17], initially, adaptive distributed extended Kalman filtering (ADEKF) is used to monitor the mobility goal.

The enhanced squid game optimizer (ISGO) assists in optimizing the ADEKF parameters to increase the mobility target tracking performance. The best course for the mobile node is then anticipated during the target movement prediction phase, which is carried out with the use of input factors, like Angle of Arrival (AoA) and Received Signal Strength (RSS).

In [18], a novel approach known as the NPO+ANN algorithm is presented to address the shortcomings of the conventional approach and increase the precision of target localization and tracking. It is based on Nomadic People Optimizer (NPO) and ANN. For node localization in WSN, [19] presents an improved whale optimization technique, which is a sophisticated metaheuristic algorithm based on the siege mechanism (SWOA). By using the SWOA method, the localization strategy also allocates the location data to unknown devices with the modeled goal function.

For indoor target localization and tracking, [20] employs the particle swarm optimization (PSO) method with the RSSI channel model. This research examines the performance of eight distinct approach combinations for target localization and tracking, including random or regular points, fixed or adaptive weights, and the region segmentation method (RSM). In [21], the rat swarm optimizer (RSO) algorithm is created. It performs competitively and yields results that are remarkably distinct from those of existing metaheuristic algorithms. In this study, the authors present the node localization issue in WSNs based on modified rat swarm optimizers (MRSOs). Localization error is further decreased in [22] in which the Range-Based Whale Optimization Algorithm (WOA) assesses the valuable position of the sensors. In this study, the WOA is used to lessen the localization mistake. The reproduction findings show that the intended methodology's presenting measures outperform the existing approach in terms of mistake-free limiting and inclusion of restrictions.

Two variations of the bat optimization algorithm (BOA) are presented in [23] to solve the limitations of the original BOA, such as getting stuck in the local optimal solution, and to more effectively localize the sensor nodes. By improving local and global search, BOA versions 1 and 2 change the exploration and exploitation of the original BOA. Multiple simulations with different target and anchor nodes test BOA versions 1 and 2. Results are compared to BOA and other optimization methods used to tackle the node localization problem. FOA-L (Fruit Fly Optimization Algorithm for node's Localization), an enhanced localization technique for WSNs, is described in [24]. This method uses FOA to reduce the difference between the estimated and real locations of unknown sensors. The suggested localization method assigns random distance and direction values to a group of flies in the search zone. The target node's location is approximated by fitness-identifying the flies with the most smell. The study [25] provides two hybrid localization techniques for localization: PSO-BPNN (Back-propagation neural network optimized by particle swarm optimization) and ELPSO (Ensemble learning particle swarm optimization).

Additionally, by using simulations, the accuracy of error optimization has been evaluated among those approaches. The proposed methods often provide a higher localization accuracy than the traditional algorithms found in the review. From the above survey, it can find that those methods offer improved accuracy but could face challenges with the complexity of ensemble learning and real-time performance. Overall, these models highlight the ongoing need for advancements in scalability, computational efficiency, and robustness in WSN target localization and tracking.

## Method details

### Proposed methodology

**Fig. 1:**
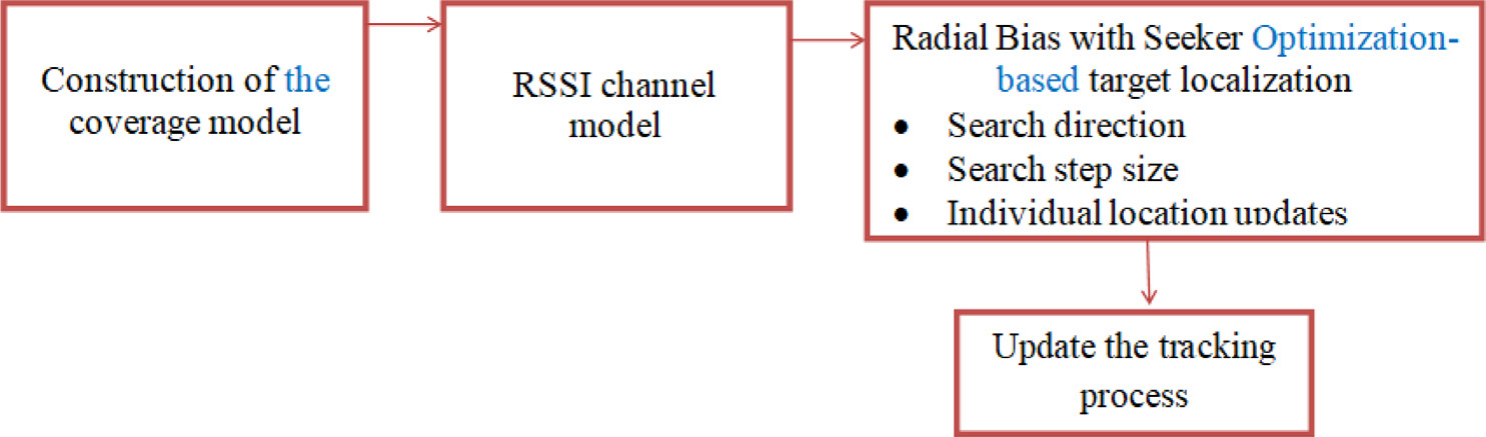
Block Diagram for Target Localization.

To enable the effective deployment of sensor nodes across a complex three-dimensional surface model, a coverage model is initially developed to ensure maximum area coverage. Alongside this, a channel model based on the Received Signal Strength Indicator (RSSI) is created to estimate the signal strength at the receiver in the absence of any obstructions or line-of-sight impediments between the transmitter and receiver. Following the development of these models, Radial Bias with Seeker Optimization (RBSO) is employed to achieve optimal target localization and tracking of data within the WSN, as illustrated in Fig. 1.

### Coverage model

**Fig. 2:**
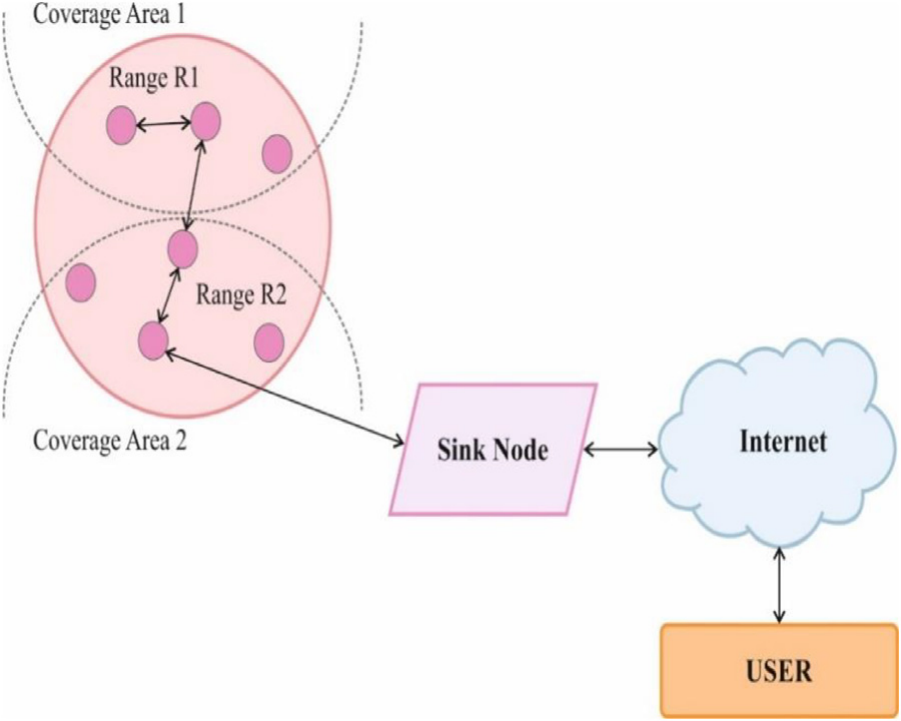
Coverage Model in WSN.

Every individual node must be deployed with a restricted sensing radius, and each SN is capable of detecting and locating objects only inside its designated sensing radius, as seen in Fig. 2. Applying detection inside the range of its sensors is a feasible way to address the issue of coverage.

Given a two-dimensional (2D) monitoring region of $W\times Lm^{2}$, a Wireless Sensor Network (WSN) is deployed with M randomly placed nodes. Let S be a collection of nodes, indicated as $S=\{S_{1},S_{2},\ldots .S_{i},\ldots S_{M},i=1,2,\ldots M$, where the coordinates of each SN, $S_{i}$, are represented as $\left(x_{i},y_{i}\right)$.

The sensing range of anSN is a circular area with a radius of $R_{s}$, centered at the node. The assumed paradigm for a two-dimensional WSN monitoring area network is as follows.

The sensor nodes have a sensing radius, denoted as $R_{s}$, and a communication radius, denoted as $R_{c}$. Both radii are specified in meters, with the condition that $R_{c}\geq R_{s}$.

The sensor nodes possess the ability to communicate, possess an adequate amount of energy, and have the capability to access time and data.

The SNs possess identical characteristics, construction, and communication capabilities.

The SNs can move unrestrictedly and promptly update their position information.

Consider a collection of target monitoring points, denoted as T, where $T=\{T_{1},T_{2},\ldots ..T_{j},\ldots T_{n}\},j=1,2,\ldots .n$. Each $T_{j}$ has coordinates $\left(x_{j},y_{j}\right)$ in the two-dimensional monitoring region of a WSN.

The target monitoring point $T_{j}$ is covered by SNs, if the distance between them is within the sensing radius $R_{s}$. Euclidean distance is the distance between SN, $S_{i}$, and target monitoring point $T_{j}$,

(1) $$d\left(S_{t},T_{j}\right)=\sqrt{{\left(x_{i}-x_{j}\right)}^{2}+}{\left(y_{i}-y_{j}\right)}^{2}$$

where $d(S_{t},T_{j})$represents the distance between node $S_{i}\left(x_{i},y_{i}\right)$to node $T_{j}\left(x_{j},y_{j}\right)$.

The node sensing model sets the probability p of the target being set to 1 based on the sensing radius $R_{s}$ and the distance $d(S_{i},T_{j})$. If $R_{s}$ is larger than or equal to $d(S_{i},T_{j})$, p is set to 1; otherwise, it is set to 0.

The expression is as follows.

(2) $$p\left(S_{i},T_{j}\right)=\left\{ \begin{array}{c} 1,\;R_{s}\geq d\left(S_{i},T_{j}\right)\\ R_{s}<d\left(S_{i},T_{j}\right) \end{array}\right.$$

where the variable $p(S_{i},T_{j})$ represents the probability of a connection between the SN, $S_{i}$, and the target monitoring point $T_{j}$.

The sensor nodes may collaborate by influencing the neighboring nodes within the installed two-dimensional WSN monitoring region. The chance of monitoring the target point $T_{j}$, when it may be covered by several sensors at the same time, is determined using the following formula.

(3) $$P\left(S,T_{j}\right)=1-\prod_{j=1}^{M}\left(1-p\left(S_{t},T_{j}\right)\right)$$

The coverage rate is the ratio of the monitoring region's total area covered by SNs to its whole area. The probability ratio to the network's surface 2D WSN monitoring area calculates the coverage ratio:

(4) $$cov_{R}=\frac{\sum_{j=1}^{M}P\left(S,T_{j}\right)}{W\times L}$$

in which the variable $cov_{R}$ represents the coverage ratio of the WSN nodes in the region where the target point is located. $P(S,T_{j})$ denotes the probability of the target point being reached by a detected node for monitoring. W×L represents the total area covered by the 2D network surface.

### RSSI channel model

It is assumed that the beacon signal RSSI may be represented by one of two models to correctly mimic the radio propagation channel and account for route loss, long-term fading, and obstacle shadowing. Unobstructed or line-of-sight (LoS) signals are represented by the lognormal shadowing model, whereas obstructed or NLOS signals are represented by a channel with extra attenuation loss. Therefore, to analyze our performance, represent the RSSI in the following manner.

(5) $$LOS\;signals=P_{r}\left(d\right)=P_{r}\left(d_{0}\right)-10n\;log\left(\frac{d}{d_{0}}\right)+N\left[0,\sigma_{f}\right]$$

(6) $$NLOS\;signals=P_{r}\left(d\right)=P_{r}\left(d_{0}\right)-10nlog\left(\frac{d}{d_{0}}\right)+N\left[0,\sigma_{f}\right]+\propto$$

Here, $P_{r}\left(d_{0}\right)$ represents the received power at a reference distance $\;d_{0}$. Path loss exponent (n) is usually between 2 and $N[0,\sigma_{f}]$, a Gaussian random variable with standard deviation $\sigma_{f}$, representing long-term fading. Additionally, ∝ represents the beacon signal attenuation due to barriers.

In the channel model, it is assumed that the noise follows a Gaussian distribution. When a variable follows a Gaussian distribution, its mean value is equivalent to its average value. However, in real-world scenarios, where there may be outliers, it is advisable to use the average value to estimate the distance since it is more resistant to the influence of outliers. To get optimal performance, the distance estimation is derived by using the average value of $RSSI_{\left(k,i\right)}$as

(7) $$d{{}^{\prime }}_{k}=10^{\frac{A_{k}-RSSI_{k}}{10n_{k}}}$$

where the averaged RSSI value measured by the kth anchor, denoted as k, is provided by the following equation.

(8) $$RSSI_{k}=\frac{1}{M}\sum_{i=1}^{M}RSSI_{\left(k,i\right)}$$

## Radial bias with seeker optimization-based target localization

**Fig. 3:**
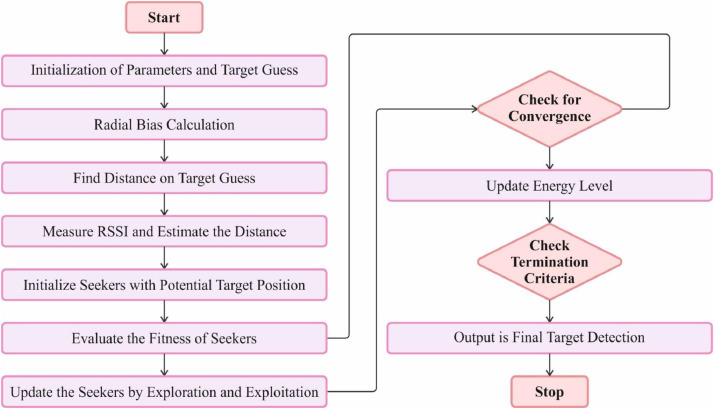
Flowchart of Radial Bias with Seeker Optimization.

The RBSO algorithm involves the random motion of each particle, which varies in direction based on its individual and collective experience. Simultaneously, it will juxtapose its encounters with those of other particles to discover an improved resolution. The RBSO algorithm has features that result in particles being influenced not just by their individual development, but also by their capacity to acquire knowledge and retain information on inter-group evolution. Moreover, the particle itself may optimize its location by making many adjustments and eventually converge to the optimal fitness value. For different applications, the particle swarm is first divided into randomly dispersed and uniformly spaced spots. Initial velocity creates a two-dimensional array of random numbers between −1 and 1. Subsequently, the fitness value of each movement is assessed, and the path with the highest fitness value is selected. This is repeated until the halting condition is reached. The estimate point is defined as the place that corresponds to the latest global optimum solution. Furthermore, the RBSO algorithm incorporates inertia weight into its target localization approach to enhance the system's efficiency. This flowchart in Fig. 3 helps visualize the sequence of operations in the RadB_SOA algorithm, illustrating the process from initialization through the iterative optimization steps to the final output of the target localization and tracking in WSNs.

### Search direction

An individual's mobility experience gradient and others' search historical location determine search direction. The directions of $f_{i,e}^{\prime }\left(t\right)$, $f_{i,a}^{\prime }\left(t\right)$, and $f_{i,p}^{\prime }\left(t\right)$ are identified as egoistic, altruistic, and preemptive, in every dimension of the ith human that may be acquired by

(9) $$f_{i,e}^{\prime }\left(t\right)=p{{}^{\prime }}_{i,best}-x_{i}^{\prime }\left(t\right)$$

(10) $$f_{i,a}^{\prime }\left(t\right)=g{{}^{\prime }}_{i,best}-x_{i}^{\prime }\left(t\right)$$

(11) $$f_{i,p}^{\prime }\left(t\right)=x_{i}^{\prime }\left(t1\right)-x_{i}^{\prime }\left(t2\right)$$

The searcher determines the search orientation by using a random weighted average approach.

(12) $$f_{i}^{\prime }\left(t\right)=sign\left(\omega {f^{\prime }}_{i,e}\left(t\right)\varphi 1{f^{\prime }}_{i,e}\left(t\right)+\varphi 2{f^{\prime }}_{i,a}\left(t\right)\right)$$

With $t1,t2\epsilon \left\{t,t-1,t-2\right\},{x^{\prime }}_{i}\left(t1\right)$ and ${x^{\prime }}_{i}\left(t2\right)$: best advantages of $\{{x^{\prime }}_{i}\left(t-2\right),{x^{\prime }}_{i}\left(t-1\right),{x^{\prime }}_{i}\left(t\right)$individually, $g_{i,best}$: historical ideal position in the i th search factor's neighborhood, $p_{i,best}$: from the ith search factor to the present locality, the weight of inertia yields the ideal location ($\delta_{1}$ and $\delta_{1}$: random integer in [0,1]).

#### Search step size

The RBSO approach pertains to the logical basis of the fuzzy approximation capability. The RBSO approach, used in computer programming, emulates some human natural languages to replicate the search patterns of human intelligence. The technique adapts most effectively to approximation objective optimization problems if it is a fuzzy rule. It is essential to extend the search step length. However, in the context of fitness, a decreased step length results in a proportional decrease in size. The search step measurement is described using the Gaussian distribution function.

(13) $$\mu \left(\alpha \right)=e^{-\frac{\alpha^{2}}{2\delta^{2}}}$$

α and β are parameters used to define the membership function. Using the Eq. (13), the probability of the output variable being outside the range of [-δ,+β] is <0.0111. Thus, $\mu_{min}$ is 0.0111. Generally, the most favorable position for an individual is characterized by a maximum value of $\mu_{max}$=1.0, while the least favorable position is represented by 0.0111. Nevertheless, to expedite the speed at which convergence occurs and get the most favorable outcome, this research establishes a value of 0.9 for the unknown step size $\mu_{max}$. The fuzzy variable function is chosen with a ``small'' target function:

(14) $$\mu_{i}=\mu_{max}-\frac{s-I_{i}}{s-I}\left(\mu_{max}-\mu_{min}\right),i=1,2,\ldots s$$

(15) $$\mu_{ij}=rand\left(\mu_{i},1\right),j=1,2,\ldots D$$

With $\mu_{ij}$, Eqs. (14) and (15) determine the count of individuals' sequence $X_{i}\left(t\right)$, arranged by function value and function $rand(\mu_{i},1)$ from high to low: real number in any partition $\left[\mu_{i},1\right]$. Eq. (14) models human random search. Eq. (15) measures the j-dimensional search interspace step.

(16) $$\propto_{ij}=\delta_{ij}-\sqrt{-ln\left(\mu_{ij}\right)}$$

With $\delta_{ij}$, the Gaussian distribution function parameter is determined by Eq. (16).

(17) $$\omega =\left(iter_{max}-t\right)/iter_{max}$$

(18) $$\delta_{ij}=\omega^{*}abs\left({x^{\prime }}_{min}-{x^{\prime }}_{max}\right)$$

where ω is the weight of the inertia. The function $x{{}^{\prime }}_{max}$represents the lowest and maximum values of the function, respectively. With each incremental advancement in the evolutionary algebra, the value of ω decreases linearly from 0.9 to 0.1.

### Individual location updates

After determining the individual's scout direction and step measurement, Eq. (19) explains the location update.

(19) $$x_{ij}\left(t+1\right)=x_{ij}\left(t\right)+\propto_{ij}\left(t\right)f_{ij}\left(t\right),i=1,2,\ldots s;j=1,2,\ldots D$$

Assigning i: i th searcher, j to individual dimension, $f_{ij}\left(t\right)$ and $\propto_{ij}\left(t\right)$ to search direction, search step size, and $x_{ij}\left(t\right)$ as well as $x_{ij}\left(t+1\right)$ to search site at t and *t* + 1, respectively.

### Process involved in the radb_soa technique

The most common use of the RadB_SOA localization technique in WSNs is to pinpoint the exact locations of the sensors. The objective is to minimize the objective function while determining the target nodes' coordinate positions. The procedures included in the RBSO method are outlined below.

Within the sensing field, M anchor nodes and N unknown nodes are randomly initialized, each with a transmission radius of R. Each anchor node ascertains its position and sends the coordinate coordinates to the nodes that are nearby. Each iteration concludes with a node that remains stable, known as a reference node. This reference node serves as the anchor node in the following iterations.

A node is considered localized if there are three or more anchor nodes located within the range of its transmission.

Additive Gaussian noise is included in the computation and in modifying the distance between the anchor and target nodes. The target node calculates the distance using $d{{}^{\prime }}_{i}=d_{i}+n_{i}$, where $d_{i}$is the actual distance between the target node's position (x,y) and the beacon's location (xi,yi), as calculated by Eq. (20).

(20) $$d_{i}=\sqrt{{\left(x-xi\right)}^{2}+{\left(y-yi\right)}^{2}}$$

where ni represents the noise that affects the calculated distance, which is within the range of $d_{i}\pm d_{i}\frac{p_{n}}{100}$. Here, $p_{n}$ is used to describe the noise ratio in the anticipated distance.

When a node's transmission range includes three anchor nodes, it is said to be localizable. The sine or cosine trigonometric formulas may be used to determine the target nodes' coordinate positions.

The target node's coordinate coordinates (x,y) that minimize the localization error are determined using the RBSO technique. The localization error is computed using the mean square distance among the target and anchor nodes, which has been reduced using Eq. (21).

(21) $$f^{\prime }\left(x,y\right)=\frac{1}{N}\left(\sum_{i=1}^{N}\sqrt{{\left(x-xi\right)}^{2}+{\left(y-yi\right)}^{2}-d{}^{\prime }}\right)$$

where N refers to the count of anchor nodes that exist within the transmission rangeN≥3.

At the end of the iteration, the optimum measure (x,y) is computed using the RBSO model.

The overall localization error is computed with the estimate of the localizable target node $N_{L}$. The validation evaluation is based on the average square of the distance between the calculated node coordinate points (Xi,Yi)and the source node coordinate points (xi,yi).

(22) $$E_{L}=\frac{1}{N1}\sum_{i=1}^{N}\sqrt{{\left(xi-Xi\right)}^{2}+{\left(yi-Yi\right)}^{2}}$$

Until the target nodes' location is ascertained, procedures 2 through 5 are repeated. The localization model relies on two key metrics: the high localization error $E_{1}$ and the count of unlocalized nodes $N_{NL}$, which is calculated as $N_{NL}=M-N_{L}$. The minimum score of $E_{1}$ and $N_{NL}$ leads to effective localization performance.

### Pseudo code of the radb_soa

Initialization of N sensor nodes
Set the initial energy level
Initialize target position and estimate target guess
Calculation of radial bias calculation (node position, target guess)
Calculate radial distance form node position to target position
Measurement of RSSI and distance estimation
For each sensor node i
Estimate the distance $\left(d_{est}\right)$ usingpath loss model
{
  $$d_{est}=10^{\frac{pt}{pr}}$$
}

Compute the biased distance using radial bias distance

Initialization of seeker optimization algorithm

 Incorporate population size and random seekers

Evaluate fitness of seekers

 Calculate the fitness based on difference between estimated and actual distance

Identify the seeker with the best fitness (lowest error)

Check the iteration and update the energy level

Indicate the termination criteria

 If the estimated target position is within an acceptable error or energy constraints are met.

 Otherwise update the target guess with the best seeker position.

Return the final estimated target position

Report the remaining energy levels of sensor nodes

Output the number of iterations taken to coverage

End the process

### Method validation

**Experimental Setup-** started with the average localization error (measured in meters) and used computer simulations to examine how the number of sensors affects localization accuracy in various schemes. The five localization options were simulated using MATLAB R2017a and a typical system model. Three network-area obstacles were simulated. A lognormal fading factor of σf 1 dB was utilized for LoS and NLoS transmissions. To allow for non-line-of-sight beacon broadcasts, included an additional attenuation factor (α) ranging from 1 to 5 dB. The barriers remained constant during all simulations, while the fading variable was simulated randomly. The results were averaged from 10 runs per circumstance and the same is shown in Table 1.

**Table 1:** Simulation Parameter.

Parameters	Values
Number of Sensor Nodes	500
Deployment Area	1000×1000 m^2^
Target Position	Dynamic
Transmission Power	0.1W
Path Loss Exponent	2.0 for Free Space
Communication Range	50 m
RSSI Measurement Rate	2dBm

The localization performance of the Radial Bias with Seeker Optimization (RadB_SOA) approach is examined in this section. The investigation involves comparing the RadB_SOA with other existing techniques, including SWOA[19], MRSO[21], and BOA[23]while adjusting the quantities of particles and Anchor Nodes (AN). The parameters considered are transmission error (TE), ranging error (RE), localization coverage, packet delivery ratio, and energy consumption.

Transmission error (TE)-The transmission error parameter typically represents the uncertainty or noise in the measurements used for localization. This parameter directly affects the accuracy of the estimated position of the target. The distance between a target and a sensor node is often estimated based on Received Signal Strength (RSS), Time of Arrival (TOA), or other metrics. However, these measurements are subjected to errors due to various factors like multipath propagation, interference, or sensor inaccuracies. The distance $d_{i}$among the i th sensor node and the target can be modeled as

(23) $$d_{i}=d_{i},\text{true}+\varepsilon_{i}$$

where $d_{i},\text{true}$is the true distance, and $\varepsilon_{i}$is the transmission error parameter, often modeled as a random variable (e.g., Gaussian noise with mean zero and variance).

**Fig. 4:**
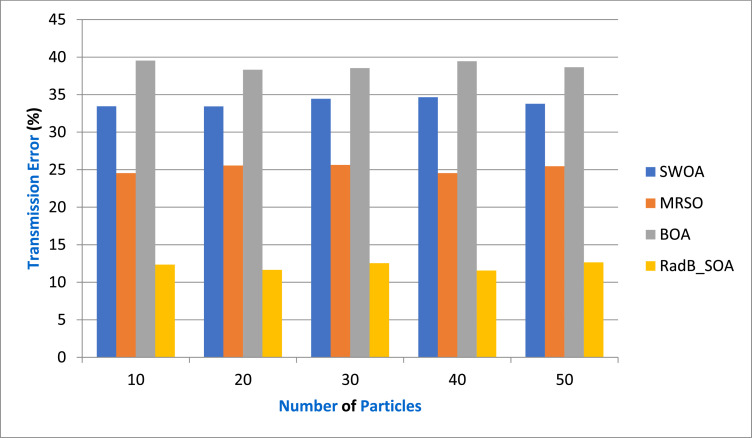
Analysis of Transmission Error.

Table 2 provides the Comparison of Transmission Error and Fig. 4 depicts the transmission error in terms of various particles by comparing it with existing SWOA, MRSO, BOA, and the proposed RadB_SOA. When compared, existing methods achieve 34.42 %, 25.65 %, and 39.65 % of transmission error, while the proposed method achieves 12.4 % of transmission error, which is 20.02 %, 13.25 %, and 27.25 % better than the aforementioned methods.

**Table 2:** Comparison of Transmission Error.

No. of Particles	SWOA	MRSO	BOA	RadB_SOA
10	33.45	24.54	39.54	12.35
20	33.43	25.56	38.31	11.65
30	34.45	25.64	38.54	12.54
40	34.65	24.54	39.45	11.56
50	33.78	25.46	38.65	12.65

Ranging error – The ranging error formula typically represents the deviation between the measured distance and the actual (true) distance between a sensor and a target. In mathematical terms, the ranging error can be modeled as

(24) $$\varepsilon_{i}=d_{i}-d_{i},\text{true}$$

where $\varepsilon_{i}$ is the ranging error for the ith measurement $d_{i}$, and trueis the actual distance between the sensor and the target.

**Fig. 5:**
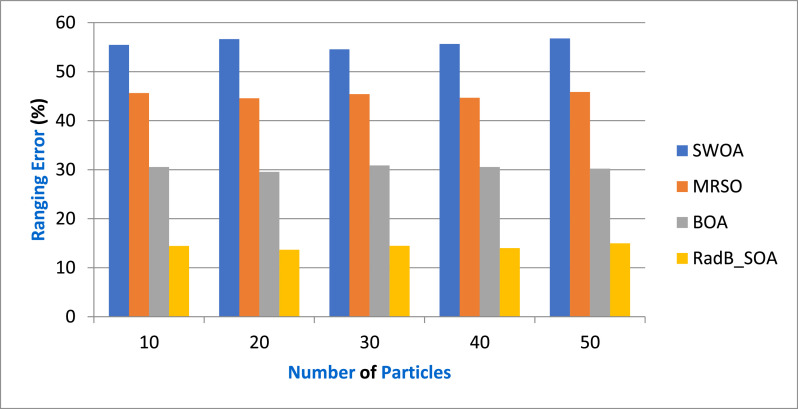
Analysis of Ranging Error.

Table 3 gives the Comparison of Ranging Error and Fig. 5 depicts the ranging error in terms of various particles by comparing it with existing SWOA, MRSO, BOA, and the proposed RadB_SOA. When compared, existing methods achieve 56.45 %, 45.32 %, and 30.67 % of ranging error, while the proposed method achieves 14.6 % of ranging error, which is 41.85 %, 30.72 %, and 16.07 % better than the aforementioned methods.

**Table 3:** Comparison of Ranging Error.

No. of Particles	SWOA	MRSO	BOA	RadB_SOA
10	55.46	45.65	30.54	14.45
20	56.65	44.56	29.56	13.67
30	54.56	45.43	30.86	14.46
40	55.65	44.66	30.56	13.99
50	56.78	45.87	30.21	14.98

Localization coverage – The localization coverage CCC can be defined as the probability that a target within a certain area can be localized with an error less than a specified threshold. Mathematically, this can be expressed as

(25) $$C=P\left(E\leq \varepsilon_{\text{max}}\right)$$

where Cis the localization coverage, Eis the localization error, and $\varepsilon_{\text{max}}$ is the maximum allowable error for the localization to be considered accurate.

**Fig. 6:**
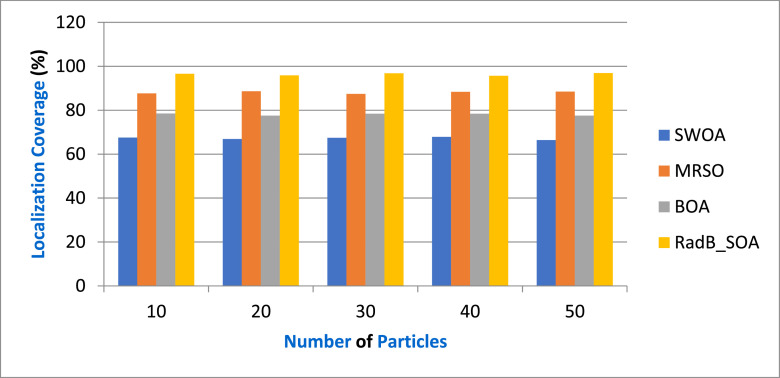
Analysis of Localization Coverage.

Table 4 gives the Comparison of Localization Coverage and Fig. 6 depicts the localization coverage in terms of various particles by comparing it with existing SWOA, MRSO, BOA, and the proposed RadB_SOA. When compared, existing methods achieve 67.86 %, 87.45 %, and 78.43 % of localization coverage, while the proposed method achieves 96.3 % of localization coverage, which is 29.56 %, 9.12 %, and 16.3 % better than the aforementioned methods.

**Table 4:** Comparison of Localization Coverage.

No. of Particles	SWOA	MRSO	BOA	RadB_SOA
10	67.54	87.65	78.54	96.56
20	66.89	88.65	77.57	95.89
30	67.43	87.45	78.43	96.78
40	67.89	88.34	78.43	95.67
50	66.43	88.46	77.56	96.89

Packet delivery ratio – It is the successful transmission ratio of packets from the original node to the target node in the network.

(26) $$\text{PDR}=\;\frac{\text{number}\;\text{of}\;\text{packet}\;\text{received}\;\text{succesfully}}{\text{Total}\;\text{number}\;\text{of}\;\text{packets}\;\text{forwarded}}$$

**Fig. 7:**
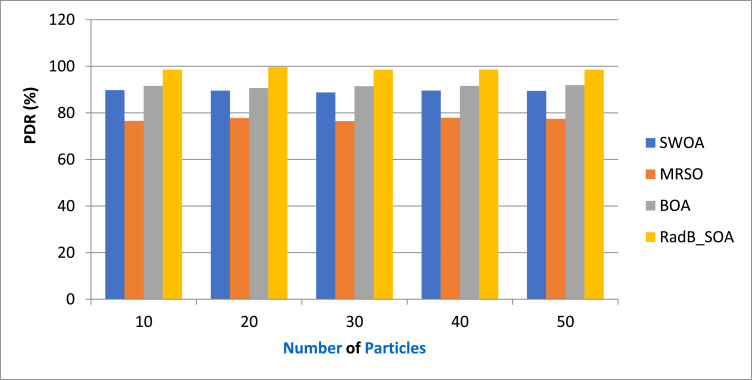
Analysis of PDR.

Table 5 gives the Comparison of PDR and Fig. 7 depicts the PDR in terms of various particles by comparing it with existing SWOA, MRSO, BOA, and the proposed RadB_SOA. When compared, existing methods achieve 89.54 %, 76.57 %, and 91.46 % of PDR, while the proposed method achieves 98.65 % of PDR, which is 9.11 %, 22.12 %, and 6.21 % better than the aforementioned methods.

**Table 5:** Comparison of PDR.

No. of Particles	SWOA	MRSO	BOA	RadB_SOA
10	89.76	76.56	91.56	98.56
20	89.54	77.76	90.65	99.65
30	88.76	76.45	91.46	98.54
40	89.56	77.89	91.56	98.56
50	89.43	77.43	91.89	98.54

Energy consumption – This is calculated as the sum of the energy of each hop.

(27) $$\text{Energy}=\;\frac{1}{p}\sum_{n}^{p}E_{n}$$

where E_n_ is the energy of the n^th^ hop, and p indicates the hops in the multihop routing.

**Fig. 8:**
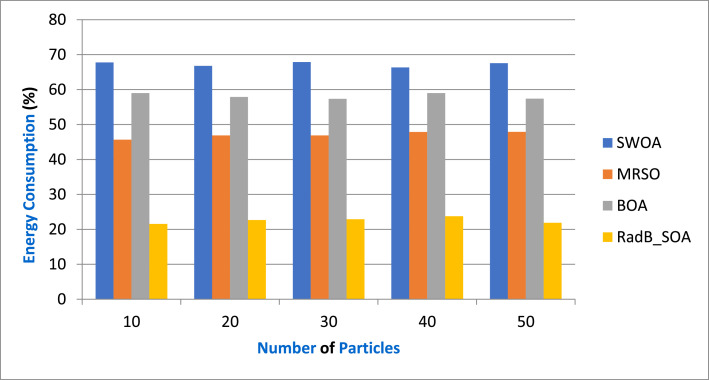
Analysis of energy consumption.

Table 6 gives the Comparison of Energy Consumption and Fig. 8 depicts the energy consumption in terms of various particles by comparing it with existing SWOA, MRSO, BOA, and the proposed RadB_SOA. When compared, existing methods achieve 67.53 %, 45.78 %, and 58.34 % of energy consumption, while the proposed method achieves 21.56 % of energy consumption, which is 45.67 %, 22.12 %, and 34.56 % better than the aforementioned methods.

**Table 6:** Comparison of Energy Consumption.

No. of Particles	SWOA	MRSO	BOA	RadB_SOA
10	67.75	45.67	58.98	21.56
20	66.78	46.87	57.89	22.65
30	67.89	46.89	57.34	22.89
40	66.34	47.87	58.98	23.765
50	67.54	47.89	57.42	21.89

In summary, the Table 7 shows the Overall Comparative Analysis. RadB_SOA is the top-performing technique, showing the lowest transmission and ranging errors, the highest localization coverage and packet delivery ratio, and the least energy consumption. SWOA, MRSO, and BOA fall behind in various metrics, with SWOA having the highest transmission and ranging errors and the highest energy consumption, while the RadB_SOA clearly stands out as the most reliable and efficient option.

**Table 7:** Overall comparative analysis.

Parameters	SWOA	MRSO	BOA	RadB_SOA
Transmission Error (%)	34.42	25.65	39.65	12.4
Ranging Error (%)	56.45	45.32	30.67	14.6
Localization Coverage (%)	67.86	87.45	78.43	96.3
Packet Delivery Ratio (%)	89.54	76.57	91.46	98.65
Energy Consumption (%)	67.53	45.78	58.34	21.56

## Conclusion

The integration of Radial Bias and Seeker Optimization Algorithms (RadB_SOA) within Wireless Sensor Networks (WSNs) has demonstrated significant advancements in energy-constrained target localization and tracking. The results indicate that the RadB_SOA outperforms traditional methods in terms of minimizing transmission and ranging errors, maximizing localization coverage, enhancing packet delivery ratios, and reducing energy consumption. This optimization leads to more reliable and efficient WSN operations, which is crucial for applications requiring precise and sustainable monitoring. For future work, further exploration into adaptive and dynamic versions of the RadB_SOA could be beneficial, allowing the algorithm to adjust in real-time to varying environmental conditions and network demands. Additionally, integrating the RadB_SOA with machine learning techniques could enhance predictive capabilities and decision-making processes in WSNs. Expanding the application of RadB_SOA to more complex and large-scale WSN environments, as well as testing its performance in diverse real-world scenarios, will be essential steps to fully validate and refine this approach.

## Limitations

**Fixed Anchor Node Distribution**: Although the RadB_SOA method addresses some challenges associated with fixed anchor node positions, it does not completely eliminate the issue. The fixed position distribution of anchor nodes may still limit the adaptability and performance of the system in dynamic environments where anchor node placement could be more flexible.

**Energy Consumption Metrics**: The abstract states that the proposed approach achieves 21.56 % energy consumption. While this is an improvement, it does not provide a benchmark against other state-of-the-art methods. The energy consumption figure might still be relatively high, depending on the context and specific application requirements.

## Ethics statements

In this Manuscript no, human participants or animals their data or biological material, are not involved.

## CRediT authorship contribution statement

**S. Yazhinian:** Conceptualization, Methodology, Software, Formal analysis, Writing – original draft, Visualization. **S. Famila:** Supervision, Project administration, Funding acquisition, Validation, Writing – review & editing. **P. Jose:** Data curation, Software, Investigation, Writing – review & editing. **Mahendar A:** Methodology, Resources, Validation, Writing – review & editing. **Sofia R:** Software, Data curation, Visualization, Writing – review & editing.

## Declaration of competing interest

The authors declare that they have no known competing financial interests or personal relationships that could have appeared to influence the work reported in this paper.

## Data Availability

No data was used for the research described in the article.

## References

[1] Safaei M., Ismail A.S., Chizari H., Driss M., Boulila W., Asadi S., Safaei M. (2020). Standalone noise and anomaly detection in wireless sensor networks: a novel time-series and adaptive Bayesian-network-based approach. Software.

[2] Safaei M., Asadi S., Driss M., Boulila W., Alsaeedi A., Chizari H., Abdullah R., Safaei M. (2020). A systematic literature review on outlier detection in wireless sensor networks. Symmetry (Basel).

[3] Slavi sˇa Tomic. Target localization and tracking in wireless sensor networks. 2017. (include complete details).

[4] Singh Yashwant, Saha Suman, Chugh Urvashi, Gupta Chhavi (2013). Computer Modelling and Simulation (UKSim), 2013 UKSim 15th International Conference on.

[5] Dai Zhicheng, Wang Shengming, Yan Zhonghua (2012). Modelling, Identification & Control (ICMIC), 2012 Proceedings of the International Conference on.

[6] Saboor A., Ahmad R., Ahmed W., Kiani A.K., Moullec Y.L., Alam M.M. (2018). On research challenges in hybrid medium access control protocols for IEEE802.15. 6 WBANs. IEEE Sens J.

[7] Ghelardoni L., Ghio A., Anguita D. (2012). Electrical Electronics Engineers in Israel (IEEEI), 2012 IEEE 27th Convention of, pages 1–5.

[8] Rongbai Z., Guohua C. (2010). Future Computer and Communication (ICFCC), 2010 the 2nd International Conference on, volume 1, pages V1–741.

[9] He T., Krishnamurthy S., Stankovic J.A., Abdelzaher T., Luo L., Stoleru R., Yan T., Gu L., Hui J., Krogh B. (2004). Proceedings of the 2nd International Conference on Mobile Systems, Applications, and Services.

[10] Ramya K., Kumar K.Praveen, Srinivas Rao D.V. (2012). A survey on target tracking techniques in wireless sensor networks. Int. J. Computer Sci. Eng. Surv..

[11] Khan Muneeb A., Saboor Abdul, Kim Hyun-chul;, Park Heemin. (2021). A systematic review of location aware schemes in the internet of things. Sensors.

[12] Khan Muneeb A., Khan Muazzam A., Rahman Anis U., Malik Asad Waqar, Khan Safdar A. (2019). Exploitingcooperative sensing for accurate target tracking in industrial internet of things. Int. J. Distrib. Sensor Netw..

[13] Oracevic A., Ozdemir S. (2014). World Congress on Computer Applications and Information Systems (WCCAIS).

[14] Kaswan A., Nitesh K., Jana P. (2017). Energy efficient path selection for mobile sink and data gathering in wireless sensor networks. AEU - Int. J. Electr. Commun..

[15] B. Hofmann-Wellenhof, H. Lichtenegger, J. Collins, Global Positioning System. Theory and Practice; Springer: Berlin/Heidelberg, Germany, 2001.

[16] Djuknic G., Richton R. (2001). Geolocation and assisted GPS. Computer (Long Beach Calif).

[17] Ramadevi N., Subramanyam M.V., Bindu C.S. (2024). Mobility target tracking with meta-heuristic aided target movement prediction scheme in WSN using adaptive distributed extended Kalman filtering. Int. J. Commun. Syst..

[18] Tariq S.M., Al-Mejibli I.S. (2024). The unscented Kalman filter for real-time target localization and tracking in WSN using hybrid NPO-ANN method. Int. J. Intellig. Eng. Syst..

[19] Dao T.K. (2024). An optimal node localization in WSN based on siege whale optimization algorithm. Cmes-Comput. Model. Eng. Sci..

[20] Lee S.H., Cheng C.H., Lin C.C., Huang Y.F. (2023). PSO-based target localization and tracking in wireless sensor networks. Electronics (Basel).

[21] Alfawaz O., Osamy W., Saad M., Khedr A.M. (2023). Modified rat swarm optimization based localization algorithm for wireless sensor networks. Wireless Personal Commun..

[22] Shakila R., Paramasivan B. (2021). An improved range based localization using whale optimization algorithm in underwater wireless sensor network. J. Ambient. Intell. Humaniz Comput..

[23] Mohar S.S., Goyal S., Kaur R. (2022). Localization of sensor nodes in wireless sensor networks using bat optimization algorithm with enhanced exploration and exploitation characteristics. J. Supercomput..

[24] Rabhi S., Semchedine F., Mbarek N. (2021). An improved method for distributed localization in WSNs based on fruit fly optimization algorithm. Autom. Control Comput. Sci..

[25] Lakshmi Y.V., Singh P., Abouhawwash M., Mahajan S., Pandit A.K., Ahmed A.B. (2022). Improved Chan algorithm based optimum UWB sensor node localization using hybrid particle swarm optimization. IEEE Access.

